# Differentiation and Structure in *Sulfolobus islandicus* Rod-Shaped Virus Populations

**DOI:** 10.3390/v9050120

**Published:** 2017-05-19

**Authors:** Maria A. Bautista, Jesse A. Black, Nicholas D. Youngblut, Rachel J. Whitaker

**Affiliations:** 1 Department of Microbiology, University of Illinois at Urbana-Champaign, 601 S. Goodwin Ave., Urbana, IL 61801, USA; bautist2@illinois.edu; 2 Department of Geophysical Sciences, University of Chicago, 5734 S. Ellis Ave., Chicago, IL 60637, USA; black2@uchicago.edu; 3 Department of Microbiome Science, Max Planck Institute for Developmental Biology, Spemannstraße 35, 72076 Tübingen, Germany; nicholas.youngblut@tuebingen.mpg.de; 4 Carl R. Woese Institute for Genomic Biology, University of Illinois at Urbana-Champaign, 1206 W Gregory Dr, Urbana, IL 61801, USA

**Keywords:** host-virus interactions, *Sulfolobus islandicus* rod-shaped virus, archaeal virus

## Abstract

In the past decade, molecular surveys of viral diversity have revealed that viruses are the most diverse and abundant biological entities on Earth. In culture, however, most viral isolates that infect microbes are represented by a few variants isolated on type strains, limiting our ability to study how natural variation affects virus-host interactions in the laboratory. We screened a set of 137 hot spring samples for viruses that infect a geographically diverse panel of the hyperthemophilic crenarchaeon *Sulfolobus islandicus.* We isolated and characterized eight SIRVs (*Sulfolobus islandicus* rod-shaped viruses) from two different regions within Yellowstone National Park (USA). Comparative genomics revealed that all SIRV sequenced isolates share 30 core genes that represent 50–60% of the genome. The core genome phylogeny, as well as the distribution of variable genes (shared by some but not all SIRVs) and the signatures of host-virus interactions recorded on the CRISPR (clustered regularly interspaced short palindromic repeats) repeat-spacer arrays of *S. islandicus* hosts, identify different SIRV lineages, each associated with a different geographic location. Moreover, our studies reveal that SIRV core genes do not appear to be under diversifying selection and thus we predict that the abundant and diverse variable genes govern the coevolutionary arms race between SIRVs and their hosts.

## 1. Introduction

The discovery of diverse and novel viruses that infect bacteria [1,2,3] and archaea [4,5,6] has revived interest in the viruses of microbes. No longer viewed only as the tools of molecular biology [7,8], viruses are now recognized to play key roles in the environment as drivers of evolution and population structure [9,10,11]. For the most part, like bacteria and archaea, different viruses are represented by one or two variants isolated on a single host. This poses a bottleneck in studying and understanding the vast and uncharacterized “viral dark matter” uncovered by metagenomics [12,13] or predicting their impact on the environment. Acidic hot springs offer an excellent system for studying host-virus interactions because they have discrete and clearly defined boundaries in which host populations are geographically isolated and culture-independent studies have identified a scaffold of viral diversity in these low-complexity environments [14]. Furthermore, in the past few decades, a great effort has been made to study and characterize the viruses that infect the crenarchaeal species that inhabit these types of hot springs (for review see [4,5,15]), uncovering novel genes [16], unusual and exceptional virion morphotypes [17], and unique virion egress mechanisms [18,19].

SIRVs (*Sulfolobus islandicus* rod-shaped viruses) were among the first viruses to challenge the notion that most crenarchaeal viruses exist only in a non-lytic, carrier state [20], making them an excellent target to study how lytic viruses shape natural microbial communities. SIRVs belong to the *Rudiviridae* family and to date, only two SIRV isolates from Iceland, SIRV1 and SIRV2, have been characterized [20,21,22,23,24] and established as a model system for studying host-virus interactions in the laboratory [22,25,26]. The virions of SIRV2, the type member of the family, are non-enveloped, stiff rods that measure approximately 23 nm × 900 nm [20]. These viruses have linear, double-stranded DNA (dsDNA) genomes, do not integrate into the host chromosome and, like some bacterial spores, package A-form DNA [27], which could play a role in stabilizing DNA in harsh environmental conditions typical of acidic hot springs. A detailed investigation of its life cycle has revealed that SIRV2 induces massive degradation of the host chromosome and causes the formation of seven fold symmetrical pyramid-like structures on the cell membrane that disrupt the host’s S-layer and open up at the end of the infection, lysing the cell and releasing new virions [18,28].

Moreover, the genomes of all *Sulfolobus islandicus* wild isolates harbor the sequence specific CRISPR-Cas (clustered regularly interspaced short palindromic repeats–CRISPR-associated proteins) adaptive immune system [29,30] that has been demonstrated to prevent natural viral infection in *S. islandicus* [31], adding a new dimension to understanding virus-host interactions in these environments. Signatures of host-virus interactions recorded in the CRISPR repeat-spacer arrays of *S. islandicus* genomes from Russia and North America suggest an ongoing coevolutionary arms race with local viral populations, including SIRVs [32]. Yet the diversity and distribution of SIRVs has only been assessed using PCR surveys of viral coat protein sequences [33] and through metagenomic sequencing [34].

To better understand host-virus coevolutionary dynamics, here we seek to augment our current knowledge about SIRV diversity in natural environments. We isolate and characterize eight novel SIRVs from different regions within Yellowstone National Park and investigate their host range and biogeographic distribution using comparative genomics and the coevolutionary signatures recorded in the CRISPR-Cas repeat spacer arrays of *S. islandicus* hosts.

## 2. Materials and Methods

### 2.1. Environmental Sampling

Liquid samples were collected from 24 acidic hot springs in seven different regions of Yellowstone National Park, United States, between June and September 2010 and from five different hot springs from the Mutnovsky volcano in Kamchatka, Russia, in August 2010 (Table S1). A total of 50 mL of each sample was filtered through a 0.22 μm PES (polyethersulfone) filter (EMD Millipore, Billerica, MA, USA) and stored at 4 °C for transportation. The remaining unfiltered samples were transported at room temperature and used to establish enrichment cultures.

### 2.2. Enrichment Cultures of Environmental Samples

Enrichment cultures (E1) were established by inoculating 19 mL of liquid dextrin-tryptone (DT) medium [35] at pH 3.5 with 1 mL of the unfiltered environmental sample. Liquid cultures were grown aerobically in glass tubes and shaken at 78 °C, and were checked daily for up to 14 days. A total of 15 mL of turbid enrichment sample was centrifuged for 20 min at 4000 rpm and the supernatant was collected and stored at 4 °C. A second enrichment (E2) was established by transferring 1 mL of E1 into 19 mL of fresh DT medium and, if turbid after up to 14 days of incubation, was centrifuged as described above.

### 2.3. Viral Isolation and Purification

A set of 11 *S. islandicus* strains were selected to represent both space and time within Yellowstone National Park and Kamchatka (Table S2), and were used as bait hosts to screen for the presence of viruses in direct filtered environmental samples and enrichment supernatants. Ten microliters of each sample were spotted on overlays of sucrose-yeast (SY) medium [36], mixed with a 10× concentrated suspension of mid-log cells of each of the 11 bait hosts, and incubated at 78 °C, as previously described [31]. Plates were monitored for the formation of clearing zones over the course of five days. Positive samples were selected for further purification on the host they infected best (Table S2). Samples were serially diluted and plated on overlays of the bait host as previously described [31] and incubated at 78 °C for up to five days. If plaque formation was detected, an individual plaque was picked from the plate using a sterile needle and used to re-inoculate 2 mL of a mid-log culture of the bait host in DT medium. The infected culture was incubated for 48 h and filtered through a 0.22 μm PES membrane filter (EMD Millipore, Billerica, MA, USA) to remove cells. The filtrate containing virus was serially diluted and plated on the bait host and the plaque purification procedure was repeated a total of three times. Filtrates of purified plaques were visually screened by transmission electron microscopy (TEM) for the presence of a single virion morphotype. Stocks of purified virus were prepared by inoculating 500 mL of mid-log cells of each bait host with 1 mL of the plaque purified virus filtrates and at 48 h post infection (hpi), were filtered through a 0.22 μm PES membrane filter (EMD Millipore, Billerica, MA, USA) to remove cells, concentrated using Corning® Spin-X® UF 20 mL Centrifugal Concentrator (MWCO, 30,000; Corning, Inc., Corning, NY, USA), and stored at 4 °C for future experiments.

### 2.4. Virus Quantification and Cross-Infection Assays

Cultures were started from frozen stocks in 2 mL of liquid DT medium for each of the *S. islandicus* host strains. When cultures turned cloudy, they were transferred into 20 mL of DT medium in tissue culture flasks (BD Falcon, Bedford, MA, USA) and incubated without shaking. For each culture, approximately 3 × 10^8^ cells were collected from mid-log cultures by low speed centrifugation, resuspended in 500 μL of DT medium, mixed with 100 μL of each dilution (10°–10^−10^), and plated on overlays of SY medium containing 0.1% yeast extract and 0.2% sucrose, as previously described [36], to determine viral titers. Dilutions were performed and plated in triplicate for each sample. SIRV stocks were diluted to 1 × 10^7^, 1 × 10^6^ and 1 × 10^4^ pfu (plaque forming units)/mL, and lawns of host cells were spotted with 10 μL of each of the virus dilutions and incubated at 78 °C. Lawns were monitored every 24 h for five days. All SIRVs were spotted on their susceptible isolation host as a positive control of infection. Three independent virus dilutions from the same original stock were spotted on three independent host cultures.

### 2.5. DNA Extraction

100× TE (Tris-EDTA) buffer was added to 15 mL of concentrated virus sample to a final concentration of 1.4× and mixed thoroughly. Samples were then treated with nucleases (RNase and DNase) prior to nucleic acid extraction, as previously described [37]. Proteinase K and SDS (sodium dodecyl sulfate) were added to a final concentration of 0.4 mg/mL and 0.1%, respectively, and incubated for 1 h at 56 °C, inverting the tubes every 10 min. Phenol extraction was performed twice using warm (60 °C) TE saturated phenol, as previously described [37], followed by an extraction with phenol/chloroform/isoamyl alcohol (24:1) at room temperature. Two and a half volumes of ice-cold ethanol and 1:50 volume of 7.5 M sodium acetate were added to the aqueous phase. The tubes were incubated overnight at −20 °C and then centrifuged for 30 min at 8000 rpm. DNA pellets were resuspended in 1× TE and desalted using QIAEX II (Qiagen, Germantown, MD, USA) beads, following the manufacturer’s instructions.

### 2.6. Genome Sequencing and Assembly

Genomic libraries were prepared for all viruses using the NexteraXT kit (Illumina, San Diego, CA, USA), following the manufacturer’s instructions. Libraries were pooled and sequenced using paired-end MiSeq v.2.5 (Illumina, San Diego, CA, USA) by the W. M. Keck Center for Comparative and Functional Genomics at the University of Illinois at Urbana-Champaign. Reads were quality filtered using the FASTX-Toolkit [38] and adapters were trimmed using Cutadapt [39]. Assemblies were performed with Geneious version 7.0 and A5-miseq [40], yielding near complete sequences excluding the terminal inverted repeats. Paired-end reads and the average insert length obtained from the high sensitivity bioanalyzer results (Agilent Technologies Inc., Santa Clara, CA, USA) of the libraries were used to enhance assemblies.

### 2.7. Comparative Genomics and Phylogenetic Analysis of SIRVs

Open reading frames (ORFs) in the newly assembled genomes of SIRV11 (GenBank accession number: KY744234), SIRV4 (GenBank accession number: KY744231), SIRV5 (GenBank accession number: KY744233), SIRV6 (GenBank accession number: KY744235), SIRV7 (GenBank accession number: KY744232), SIRV8 (GenBank accession number: KY744229), SIRV9 (GenBank accession number: KY744228), SIRV10(GenBank accession number: KY744230 and the publicly available genomes of SIRV1 (GenBank accession number: NC_004087.1), and SIRV2 (GenBank accession number: NC_004086.1) were predicted using Prodigal v2.6.1 [41]. Homologous gene clusters were identified with OrthoMCL [42] using default parameters and were manually screened using all-against-all BLAST to ensure that all matches within a cluster had a bit score/max bit score value of 0.3 or higher [43]. TBLASTN searches of the amino acid sequences in each cluster against a database with all SIRV genomes were performed to find possible ORFs that were missed or miscalled in the original genome annotations. Genes that had not been previously characterized were analyzed using the NCBIs CD-Search tool [44]. Core and variable gene clusters with five or more members were translation aligned using MUSCLE [45] and were manually curated. Maximum likelihood phylogenies of individual core and variable gene clusters, in addition to a concatenated nucleotide alignment of the 30 core genes, were calculated with MEGA v. 6.06 [46] using the best fit model with 1000 bootstrap replicates. Pn/Ps ratios were calculated using SNAP v2.1.1 [47,48] and the number of base substitutions per site between sequences were conducted with the Jukes-Cantor model using Mega v.6.06 [46]. The codon positions included were 1st+2nd+3rd+Noncoding and all positions containing gaps and missing data were eliminated.

### 2.8. Phylogenetic Analyses of S. Islandicus Strains

Twelve MLSA (multilocus sequence analysis) loci (MobA, NiTra, IsoL, PAcyl, FePer, OCycl, BGlu, Heli, NuTrs, PeProt, SeBin, and ADehy) previously selected from *S. islandicus* core genes to be evenly distributed around the genome and maximize the number of single nucleotide polymorphism (SNPs) in the Mutnovsky population [49] were extracted from published *S. islandicus* genomes [50,51] and the draft genome assembly of *S. islandicus* Y.08.82.36 [31]. Nucleotide sequences were aligned using MUSCLE [45] and were manually inspected. Phylogenies of a concatenated nucleotide alignment of the 12 MLSA loci were inferred under maximum likelihood, as described above.

### 2.9. Host Variation

Homologs of cluster sso3138-sso3141 and cluster sso2386-sso2387, encoding the cell surface and type IV secretion proteins implicated in rudivirus entry in *S. solfataricus* [26], were found in all *S. islandicus* published genomes (M.16.4 (NC_012726.1), M.16.40 (NZ_AHJQ00000000.1), LAL14/1(NC_021058.1), REY15A (NC_017276.1), HVE10/4 (NC_017275.1), Y.G.57.14 (NC_012622.1), Y.N.15.51 (NC_012623.1), L.D.8.5 (NC_013769.1), L.S.2.15 (NC_012589.1), M.16.2 (NZ_AHJK00000000.1), M.16.12 (NZ_AHJL00000000.1), M.16.22 (NZ_AHJN00000000.1), M.14.25 (NC_012588.1), M.16.27 (NC_012632.1), M.16.46 (NZ_AHJS00000000.1), M.16.13 (NZ_AHJM00000000.1), M.16.47 (NZ_AHJT00000000.1), M.16.23 (NZ_AHJO00000000.1), M.16.30 (NZ_AHJP00000000.1), M.16.43 (NZ_AHJR00000000.1)) using BLASTN. Each gene was translation-aligned using MUSCLE [45] and manually curated, and maximum likelihood phylogenies were then constructed, as described above.

### 2.10. Analysis of CRISPR Spacer Matches

A total of 4370 CRISPR spacers from all published *S. islandicus* genomes and contigs from unpublished draft genomes from three Yellowstone National Park *S. islandicus* strains (Y08.82.36, NL13.C01.02, NL01.B.C01.24) were extracted using CRISPRfinder [52] and oriented based on the repeat sequence flanking the spacer. In-house software (CLdb v0.2, a computational toolset for comprehensively analyzing CRISPR locus diversity at the population or community-level, available at https://github.com/nick-youngblut/CLdb) was used to assess the CRISPR spacer matches of host strains against SIRVs, taking into account different parameters that have been previously shown to be important for CRISPR immunity in *Sulfolobus*, such as the percentage of the entire spacer matched, the percentage of the 5′ half and seed region of the spacer matched, and the presence of a protospacer associated motif (PAM) [53].

## 3. Results

### 3.1. Sampling

A total of 137 enrichment samples from 24 springs in seven different regions of Yellowstone National Park collected between June and September 2010 and 20 samples from six springs in the Mutnovsky Volcano in Kamchatka, Russia, were screened on a panel of diverse *S. islandicus* hosts (Tables S1 and S2). A total of eight *Sulfolobus islandicus* rod-shaped viruses were successfully isolated and purified from samples from Norris Geyser Basin and Nymph Lake, where 90% of the samples in Yellowstone National Park were collected. Kamchatka samples yielded no rudiviruses.

### 3.2. SIRV Isolation and Structure

**Figure 1:**
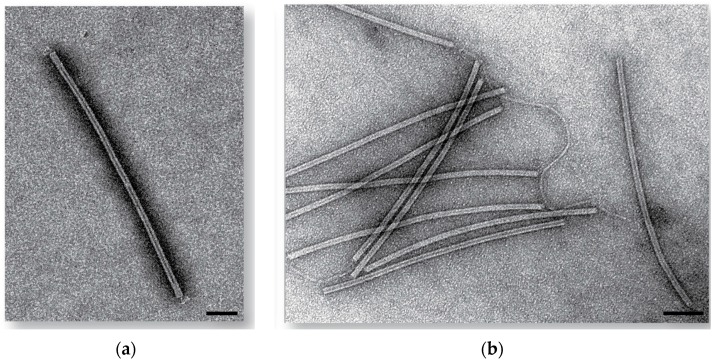
Electron Micrographs of *Sulfolobus islandicus* rod-shaped viruses SIRV5 (**a**) and SIRV8 (**b**) stained with 2% uranyl acetate. Scale bar 100 nm.

All SIRVs were plaque purified three times on the *S. islandicus* strain they initially formed a zone of clearing on and were further characterized. The plaque morphology of these viruses was clear and uniform. The virus particles for all purified SIRVs constitute flexible rods which are 879 (±59) nm long and 22 (±3) nm wide, and display short tail fibers, as previously described [21] (Figure 1 and Figure S1). We have designated these new viruses SIRV11 through SIRV10.

### 3.3. Comparative Genomics of Sulfolobus Islandicus Rod-Shaped Core Genome

**Figure 2:**
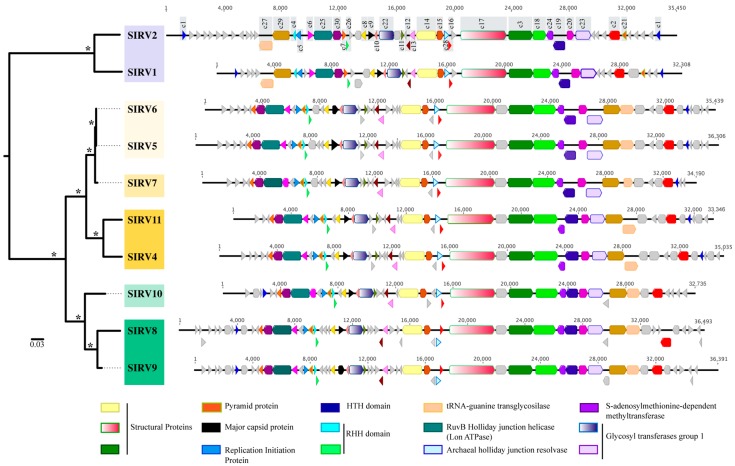
Maximum Likelihood phylogeny of a concatenated nucleotide alignment of SIRV core genes. Asterisks (*) at branches indicate 100% bootstrap support as a percent of 1000 replicates. Scale bar represents substitutions per site. Colored boxes highlight strains by geographic location (yellow: Nymph lake, green: Norris Geyser Basin and blue: Iceland). Genome maps of the SIRVs are shown to the right. Genes shown in color represent core genes and cluster numbers are denoted on the type virus SIRV2. Annotations or predicted function (if available) for core genes are shown. Variable genes are shown in gray.

The average genome size was 34,769 bp (±1545), with between 49 and 61 ORFS predicted for each of the genomes and an average G + C content of 26.5% (±0.6) (Table S3). A total of 94 homologous gene clusters from the genomes of eight Yellowstone National Park SIRVs were identified, in addition to the previously described SIRV1 and SIRV2 from Iceland. Thirty of these clusters are core (shared among all strains) and represent between 50% and 60% of the ORFs in each genome (Figure 2).

#### 3.3.1. Core Genome

Among these core genes are the major capsid protein [54], three structural proteins [55,56], and the protein responsible for the formation of pyramid-like structures that allow virion release upon infection [18,57] (Figure 2, Table S4). Other proteins important for replication initiation [23] and transcriptional regulation [24] are part of the core genome. Of the core proteins, eight clusters (c22, c23, c24, c26, c27, c28, c29, and c30) match sequences in *S. islandicus* genomes. These sequences include glycosyltransferases, methyltransferases, a tRNA-guanine transglycosilase, and DNA replication proteins (Table S4). The ratio of nonsynonymous to synonymous variation (Pn/Ps) within the 30 SIRV core clusters (Table S5) shows that all 30 clusters are under relaxed purifying selection (Pn/Ps < 1) [40,41].

The majority of core genes, including all structural proteins, the major capsid protein, glycosyltransferases, and methyltranferases, are clustered towards the central region of the genome and are syntenous in all 10 SIRVs (Figure 2). The remaining core genes are syntenous in all Yellowstone SIRVs, but are rearranged as compared to the Icelandic SIRVs (Figure 2). Homologues to some of these core proteins have been reported in other rudiviruses, in some lipothrixiviruses, and in the *Sulfolobus tengchongensis* spindle-shaped virus (Table S4) [20,24,54,55,56].

Figure 2 shows a maximum likelihood phylogeny of a concatenated nucleotide alignment of the 30 core SIRV genes where strains cluster by geographic location with significant bootstrap support (100%). Yellowstone strains not only cluster by the region within Yellowstone, but also by the spring from which they were isolated within each region. The majority (19/30) of maximum likelihood phylogenies for each individual core cluster support this grouping by geographic location (Yellowstone and Iceland) (Figure S2). Some core genes show incongruent topologies (Figure S3) with significant bootstrap support, suggesting there is recombination between viruses from different hot springs and different regions within Yellowstone. For core cluster 3 and cluster 5, some of the Yellowstone isolates were more closely related to Iceland isolates than to other Yellowstone isolates (Figure S3), suggesting either that novel alleles were gained by horizontal gene transfer in Yellowstone, diversifying selection is playing a role in promoting diversity in this region, or both. Gene transfer between Iceland and Yellowstone in terms of these SIRV viruses is also possible, but less likely. We did not observe any relationship between the genes that showed incongruent topologies and their respective, distributed locations throughout the genome.

**Figure 3:**
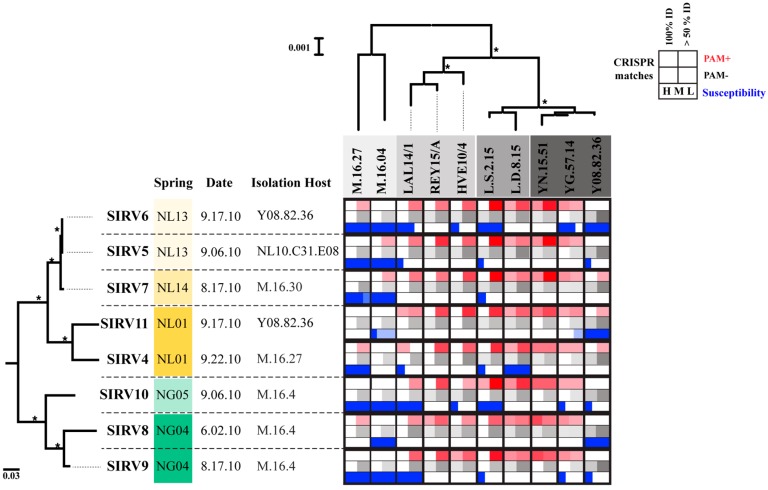
*Sulfolobus islandicus* susceptibility and CRISPR (clustered regularly interspaced short palindromic repeats) spacer matches against Yellowstone SIRVs. Blue bars indicate that a zone of clearing was formed at high (H) 1 × 10^7^ pfu (plaque forming units)/ml, medium (M) 1 × 10^6^ pfu/mL or low (L) 1 × 10^4^ pfu/mL viral challenge. Darkest blue denotes that a zone of clearing was formed on that host on all three replicates, lightest blue denotes that a plaque was formed only on one of the replicates, and white denotes that no clearing was formed in any of the replicates. Presence of CRISPR matches with a protospacer adjacent motif PAM (red) and without a PAM (grey) are shown. A darker shade represents a higher number of matches. Empty cells indicate that no matches fit the criteria. Tree above *S. islandicus* host strains represents a Maximum Likelihood phylogeny of a concatenated nucleotide alignment of 12 MLSA (multilocus sequence analysis) loci for these strains. Asterisks on branches indicate bootstrap support >80% as a percent of 1000 replicates. Scale bar represents substitutions per site.

Our data supports that the differences we observe between clades result from the evolutionary history of isolation of the viruses and not from the isolation host range or time of isolation. SIRV8, SIRV9, and SIRV10 were all isolated using the same host M.16.4 (Figure 3), yet SIRV11, SIRV5, SIRV6, and SIRV7 all infect M.16.4. SIRV6 and SIRV11 were both isolated on Y.08.82.36, a host isolated from the Norris Geyser Basin region of Yellowstone, yet they group by location and neither groups with the Norris Geyser Basin isolates. We can also observe that the viruses do not group by the date the sample was collected (Figure 3), as SIRV6 and SIRV11 come from samples collected on the same day, as do SIRV5 and SIRV10, yet they cluster by geographic location of isolation.

#### 3.3.2. Variable Genome

Variable genes for all SIRV isolates were generally clustered towards the periphery of the genome (Figure 2). The majority of variable genes have no significant matches to other proteins or known protein domains in the databases (Table S6). Two have putative annotations as a DNA binding protein [23,56] and a dUTPase [55].

**Figure 4:**
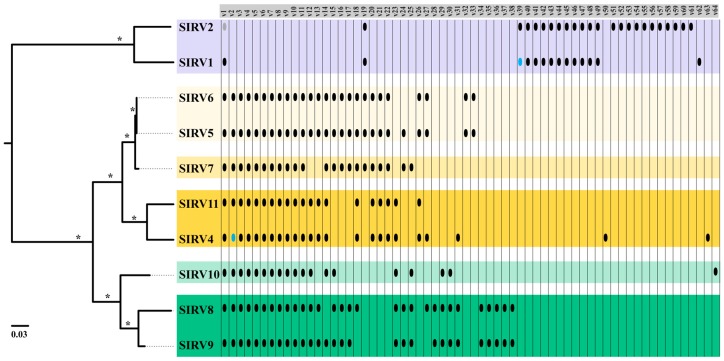
Presence/absence of variable genes in the different SIRVs. Colored boxes highlight strains by geographic location (yellow: Nymph lake, green: Norris Geyser Basin and blue: Iceland). Tree represents a Maximum Likelihood phylogeny of a concatenated nucleotide alignment of SIRV core genes. Asterisks (*) at branches indicate 100% bootstrap support as a percent of 1000 replicates. Scale bar represents substitutions per site. Grey dots represent sequence is present, but there is no start codon. Blue dots represent gene found in more than one copy.

Variable genes of SIRV also support a historical biogeographic distribution, where more closely related strains share more of their variable gene content (Figure 4). SIRV1 and SIRV2 share 11 genes that are absent from the Yellowstone SIRVs and SIRV2 has an additional set of 11 genes that are not shared with any other SIRV. A total of 36 of the variable genes present in Yellowstone SIRVs are absent from both of the Icelandic isolates. Within Yellowstone, SIRVs that are from the same hot spring share more of their variable genes (Figure 4). Three variable gene clusters are exclusive to Nymph Lake isolates, and two are unique for all Norris Geyser Basin isolates. Eight clusters are shared only among isolates that were isolated from the same hot spring. Not only the presence/absence of these variable genes is shared among isolates from the same geographic location, but also their location and organization in the genome (Figure S4). Grouping by geographic location is supported by phylogenetic analyses of variable clusters with five or more sequences (Figure S5) where there is significant bootstrap support.

### 3.4. Host-Virus Interactions Recorded in the S. Islandicus CRISPR Repeat Spacer Arrays

**Figure 5:**
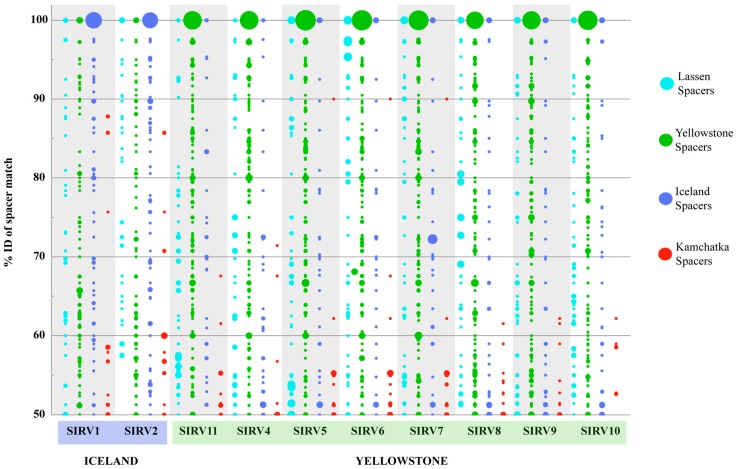
CRISPR spacer matches of spacers from *S. islandicus* strains from different geographical locations to SIRVs from Yellowstone and Iceland. Only matches with >50% identity are shown. Diameter of circles indicates the number of matches at that % identity. Smallest circle represents one match, largest circle represents 48 matches.

To further test SIRV biogeographic patterns, we looked at the signatures of host-virus interactions recorded in the CRISPR-Cas adaptive immune system, which is present in all *Sulfolobus islandicus* strain*s* sequenced to date and has been demonstrated to confer immunity against natural viral infection in this species [31]. To study biogeographic patterns, we looked at the nucleotide identity of the spacer matches to the SIRVs and we used this as a measure of divergence of the virus fragment recorded in the CRISPR array and the sequenced virus, as has been previously described [32]. All spacers were compared to the SIRV genomes and matches with more than 50% sequence identity to the entire spacer are shown (Figure 5). Figure 5 shows that spacers match with a higher percent identity to local as compared to foreign viruses, supporting that *S. islandicus* hosts have interacted with viruses that are more closely related to local virus populations. Of all the Yellowstone spacers on our database, 29.4% (225/765) matched an SIRV with more than 50% sequence ID of the entire spacer. Although the smallest pool of spacers in our database was from Lassen National Park, 18.2% (93/510) of the spacers sampled from this region matched SIRVs with more than 50% ID. On the contrary, only 2.4% (55/2330) of the spacers from Kamchatka that correspond to more than 50% of the spacers in our database matched SIRV genomes from Yellowstone or Iceland with >50% sequence identity. To further investigate this, we examined a set of spacers that were PCR amplified and sequenced from Kamchatka *S. islandicus* isolates [49], excluding those already in our previous database, to find that only 3.2% of the spacers matched any SIRV with more than 50% ID of the entire spacer, consistent with the fact that SIRVs were not recovered from Kamchatka.

Although on average 41.3% (±1.3) of core, 36.7% (±1.9) of variable, and 58.9% (±2.4) of non-coding SIRV base pairs are matched, the distribution of spacers with significant matches (>50% of the entire spacer) show that more spacers from our database match core genes (66.7 ± 2.0%) than they do variable genes (12.0 ± 6.5%) or non-coding regions (21.3% ± 6.4).

### 3.5. Host Range of SIRVs

Lawns of *S. islandicus* strains from different geographic locations were spotted with 10 μL of the eight isolated SIRVs at three different concentrations: high (1 × 10^7^ pfu/mL), medium (1 × 10^6^ pfu/mL), and low (1 × 10^4^ pfu/mL), and were monitored for the appearance of clearing zones for five days.

Figure 3 shows the differences among host populations isolated from different locations. Strains from Kamchatka for the most part lack high identity CRISPR spacers to any of the SIRV strains with or without a PAM. Kamchatka strains were susceptible to all SIRV strains. Figure 3 illustrates that these infection patterns are not correlated with the phylogeny of the viral core genes or the genes present in these strains, and therefore, are likely host-derived traits. In the Yellowstone, Lassen, and Iceland strains, there is greater potential for immunity with abundant CRISPR spacers >50% ID and containing a PAM (Figure 3, Table S7). This is correlated to an overall lower infectivity of these viruses in these strains (Figure 3). Strain Y.08.82.36 does not appear to contain significant CRISPR matches to five of the SIRVs (SIRV5, SIRV6, SIRV8, SIRV9, and SIRV10), but is not infected by three of these strains (SIRV5, SIRV9, and SIRV10) except at the highest titer challenge, suggesting variation in infectivity that is independent of the CRISPR immunity occurring among these viruses. Six virus-host pairs that should have immunity formed plaques, even at the low titer challenge, showing potential for anti-CRISPR activity (Y.08.82.36:SIRV11, L.D.8.5:SIRV4, LAL1/14:SIRV9, and LAL14/1-L.S.2.15:SIRV6 and 10). The viruses that can potentially evade immunity do not share a phylogenetic affiliation or clustered set of variable genes and possibly do not share a specific mechanism to target CRISPR immunity.

To further investigate the susceptibility patterns of these hosts to SIRV infection, we compared two gene loci that encode for cell surface proteins and type IV secretion proteins in *Sulfolobus solfataricus* that have been implicated in the resistance to SIRV [26] in this species. Phylogenetic analyses of these genes in *S. islandicus* hosts show clustering by geographic location (Figure S6), but no correlation to the SIRV infectivity patterns. The ratio of nonsynonymous to synonymous variation (Pn/Ps) within these genes (Table S8) shows that these genes are under relaxed purifying selection (Pn/Ps < 1) and are very conserved, even within strains from different geographic locations [40,41].

## 4. Discussion

Over the past few decades, our understanding of the breadth of viral diversity has been challenged with the development of culture-independent sequencing technologies. As the amount of sequence data accumulates at exponential rates in databases, most of these sequences do not resemble anything in them. Our efforts to decipher this vast amount of viral diversity are hindered by the few type host-virus pairs that are studied in the laboratory. We have isolated and characterized eight novel SIRVs from Yellowstone National Park and established them in culture in a genetically tractable system, thus augmenting the toolbox to understand host-virus coevolutionary dynamics in the environment and study them in the laboratory. Although these viruses share a set of 30 core genes with previously characterized SIRV1 and SIRV2 isolates, they comprise an even larger set of 64 variable genes clusters that are not shared among all isolates. Investigating and understanding the role of viral variable genes in the interaction with their hosts will shed light on “viral dark matter”, which is probably greatly composed of these diverse and abundant variable genes.

Although no genomic synteny was previously observed between members of the *Rudiviridae* [58], comparisons between members of the same virus type had not been possible due to the small sample size. The comparison of all 10 SIRVs in this study showed synteny for more than 50% of the core genes around the structural proteins. Our results support previous observations that rudiviruses accumulate variable and unique genes, the majority of which have an unknown function, at their termini, and have their core genes clustered towards the center of the genome [55], as has been observed in the eukaryotic poxviruses [59]. We observed that core genes are under relaxed purifying selection (Pn/Ps < 1). Selection is thus acting against deleterious nonsynonymous substitutions, suggesting that these genes are important for the virus life cycle and do not appear to be under the diversifying selection which would be expected for viral proteins that interact with host cells. Variable genes present in at least five of the SIRVs are also under purifying selection and we therefore predict that their variable gene gain and loss governs the coevolutionary arms race between SIRVs and their hosts. These variable genes have been shown to be acquired through recombination with other viruses, but also from their hosts [22], and might be carrying diverse genetic functions that allow the viruses to overcome their hosts’ defenses.

Our results show that *Sulfolobus islandicus* rod-shaped viruses have a biogeographic distribution at a global and local scale within Yellowstone National Park. This biogeographic pattern is consistent with what has been observed in *S. islandicus* populations around the world [35,51] and *Sulfolobus*-spindle-shaped viruses [32]. Our findings do not support previous studies which found no biogeographic pattern of SIRVs within Yellowstone National Park [33] based on the sequence of the coat protein (cluster 9), illustrating how one gene is not sufficient to resolve the viral spatial structure. This biogeographic distribution was supported by the variable gene content, where more variable genes are shared between strains from the same location. This is different to what has been previously observed with *Sulfolobus* spindle-shaped viruses, where the core genome displays a biogeographic distribution, but the variable component of the genome is not associated with local populations [32]. To further evaluate this observation, SIRVs from more distant locations need to be sampled.

On a global scale, the biogeographic pattern observed by the core genes and the distribution of variable genes is supported by the signatures of host-virus interactions recorded in the CRISPR spacers of *S. islandicus* strains. On average, 22.23% of the spacers from Yellowstone *S. islandicus* strains match SIRV genomes from Yellowstone with more than 50% identity of the entire spacer. A similar pattern was observed with Iceland spacers and SIRVs However, only 0.56% of the spacers from Kamchatka had significant matches to SIRV sequences, although we had the largest set of spacers to sample from this population. Interestingly, these few matches do not cluster in the conserved core proteins, suggesting that SIRVs from Kamchatka, if present, are very divergent from those present in Yellowstone and Iceland, or that these genes are shared between SIRV and a different virus present in Kamchatka. An alternative hypothesis is that these viruses are not predominant predators in the Kamchatka populations sampled. This is supported by the fact that we were unable to isolate this type of virus from the samples collected from this region.

Kamchatka strains show resistance (not immunity) to three of the eight SIRVs tested, suggesting that although they do not encounter SIRV, they have evolved resistance to it or to a related virus that infects using the same mechanism. We examined the only two known loci that have been shown in *S. solfataricus* to render the cells resistant to SIRV2 infection upon inactivation [26]. These genes cluster broadly by geographic location, which does not explain the susceptibility patterns observed. In the 20 *S. islandicus* hosts examined, these genes were present and appear to be under relaxed purifying selection. We would expect genes that are determining susceptibility to viruses to be under diversifying selection, yet mutations in these genes can come at significant fitness costs for the host as they are oftentimes involved in important physiological processes such as sugar intake (e.g., *LamB* is an outer-membrane porin in *Escherichia coli* that is used to transport maltose into cells and it is also the receptor of phage λ) [60]. In REY15A, sso3139 and 3140 have premature stop codons and thus might not be functional. Interestingly, this strain was not infected by SIRVs. Y.N.15.51 was also not infected by the SIRVs assayed, but in this strain, sso3139 and sso3140 are not disrupted. These results highlight the need for further studies to determine the attachment and entry mechanism of microbial viruses to better understand the factors such as immunity and resistance playing a role in virus infection.

Host-virus interactions in natural environments are a multifactorial process and we need to tease apart how these different factors might contribute to the observed patterns. CRISPR immunity against SIRVs in Kamchatka was very low and is probably not a determinant of the susceptibility patterns observed. In Yellowstone, Lassen, and Iceland, where CRISPR hits were abundant, we observed that in many instances, the presence of one or more CRISPR spacer matches resulted in no infection or infection only at a high titer, which might be overwhelming the CRISPR system. However, there are few exceptions where we have one or more perfect spacer matches with a PAM and multiple imperfect matches to the virus, where we would predict that the strain would be immune, yet the strain was still susceptible. The viruses that can evade immunity do not share a phylogenetic affiliation or clustered set of variable genes and therefore, may be related to specific host immune system targeting or other host factors such as faulty or inactivated *cas* genes. Another possibility is that SIRVs carry anti-CRISPR proteins such as those described for bacteriophages that infect *Pseudomonas aeruginosa* [61,62]. These anti-CRISPR proteins could be interfering with specific proteins of the CRISPR systems in these strains, that although they belong to the same Types (Type I and Type III), are highly diverse [63] within these strains and carry different *cas* gene cassettes.

## Supplementary Materials

The following are available online at www.mdpi.com/1999-4915/9/5/120/s1, Figure S1: Electron Micrographs of SIRVs, Figure S2: Maximum Likelihood phylogenies of individual SIRV core genes that support biogeographic distribution, Figure S3: Maximum Likelihood phylogenies of individual SIRV core genes with incongruent topologies, Figure S4: Organization of variable gene clusters on Yellowstone SIRVs, Figure S5: Maximum Likelihood phylogenies of individual SIRV variable genes with 5 or more representatives, Figure S6: Maximum Likelihood phylogenies of genes implicated in SIRV resistance in *S. solfataricus*, Table S1: Environmental sample collection, Table S2: Host strain panel for virus purification, Table S3: Length, coverage, GC content and spacer match coverage of SIRVs, Table S4: SIRV core gene clusters, Table S5: Estimates of synonymous vs. non-synonymous substitutions and evolutionary divergence between sequences of each of the 30 SIRV core clusters, Table S6: SIRV variable gene clusters, Table S7: CRISPR spacer matches of *S. islandicus* strains against SIRVs with and without a protospacer associated motif (PAM), Table S8: Estimates of synonymous vs. non-synonymous substitutions and evolutionary divergence between *S. islandicus* sequences of two loci implicated in SIRV resistance in *S. solfataricus*, Table S9: Estimates of synonymous vs. non-synonymous substitutions between sequences SIRV variable clusters with 5 or more representatives.
