# Supplementary material for: Differentiation and Structure in Sulfolobus islandicus Rod-Shaped Virus Populations
Source: Viruses. 2017 May 19;9(5):120. doi: 10.3390/v9050120 (PMC5454432; doi:10.3390/v9050120)
Supplement: Supplementary file 1 [file viruses-09-00120-s001.zip › Supplementary Materials/Figure S1.pdf]

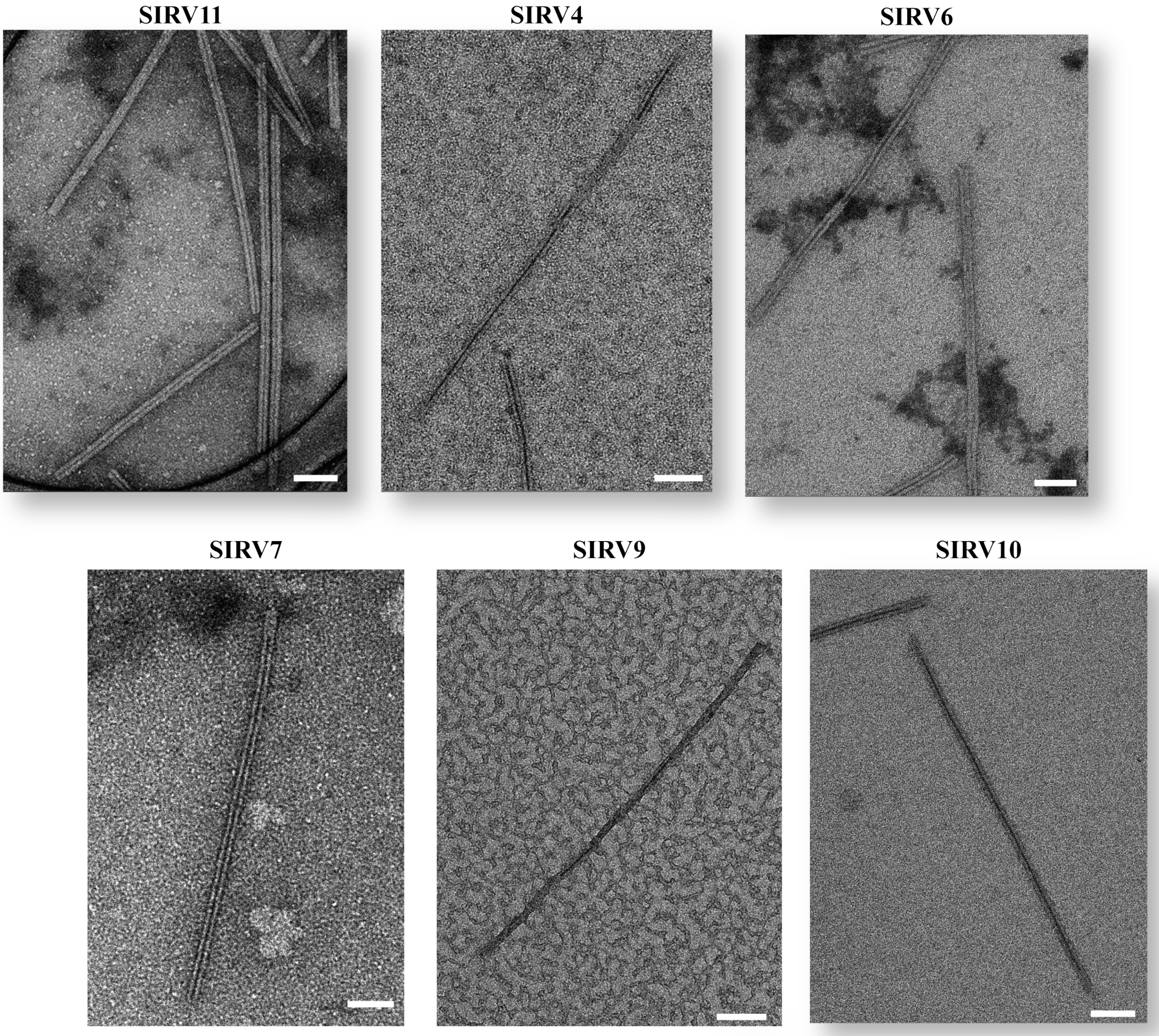

Figure S1. Electron Micrographs of SIRVs stained with 2% uranyl acetate. Scale bar 100nm.
