# Supplementary material for: Differentiation and Structure in Sulfolobus islandicus Rod-Shaped Virus Populations
Source: Viruses. 2017 May 19;9(5):120. doi: 10.3390/v9050120 (PMC5454432; doi:10.3390/v9050120)
Supplement: Supplementary file 1 [file viruses-09-00120-s001.zip › Supplementary Materials/Figure S4.pdf]

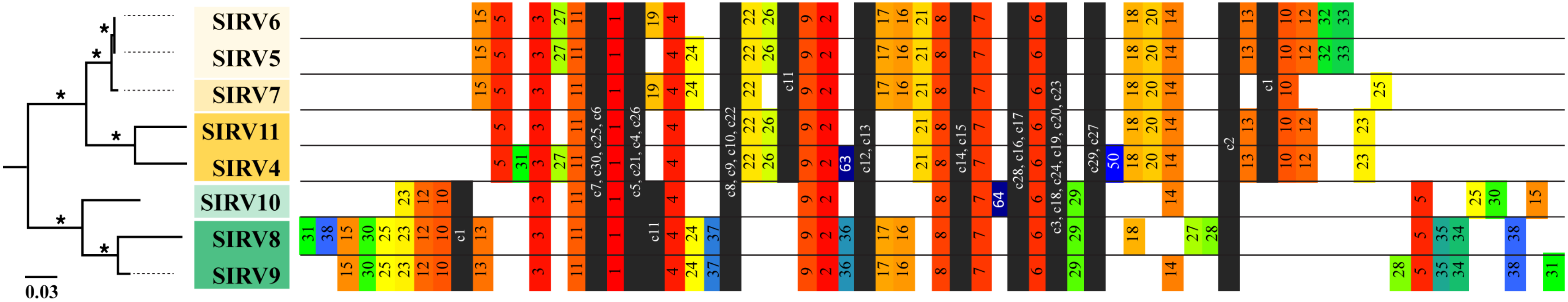

Figure S4. Organization of variable gene clusters on Yellowstone SIRVs. Variable gene clusters are shown in order and each column represents a different variable gene cluster. Numbers in boxes correspond to the variable gene cluster they represent and are colored by number. Black bars denote blocks or core genes.
