# Supplementary material for: Differentiation and Structure in Sulfolobus islandicus Rod-Shaped Virus Populations
Source: Viruses. 2017 May 19;9(5):120. doi: 10.3390/v9050120 (PMC5454432; doi:10.3390/v9050120)
Supplement: Supplementary file 1 [file viruses-09-00120-s001.zip › Supplementary Materials/Figure S6.pdf]

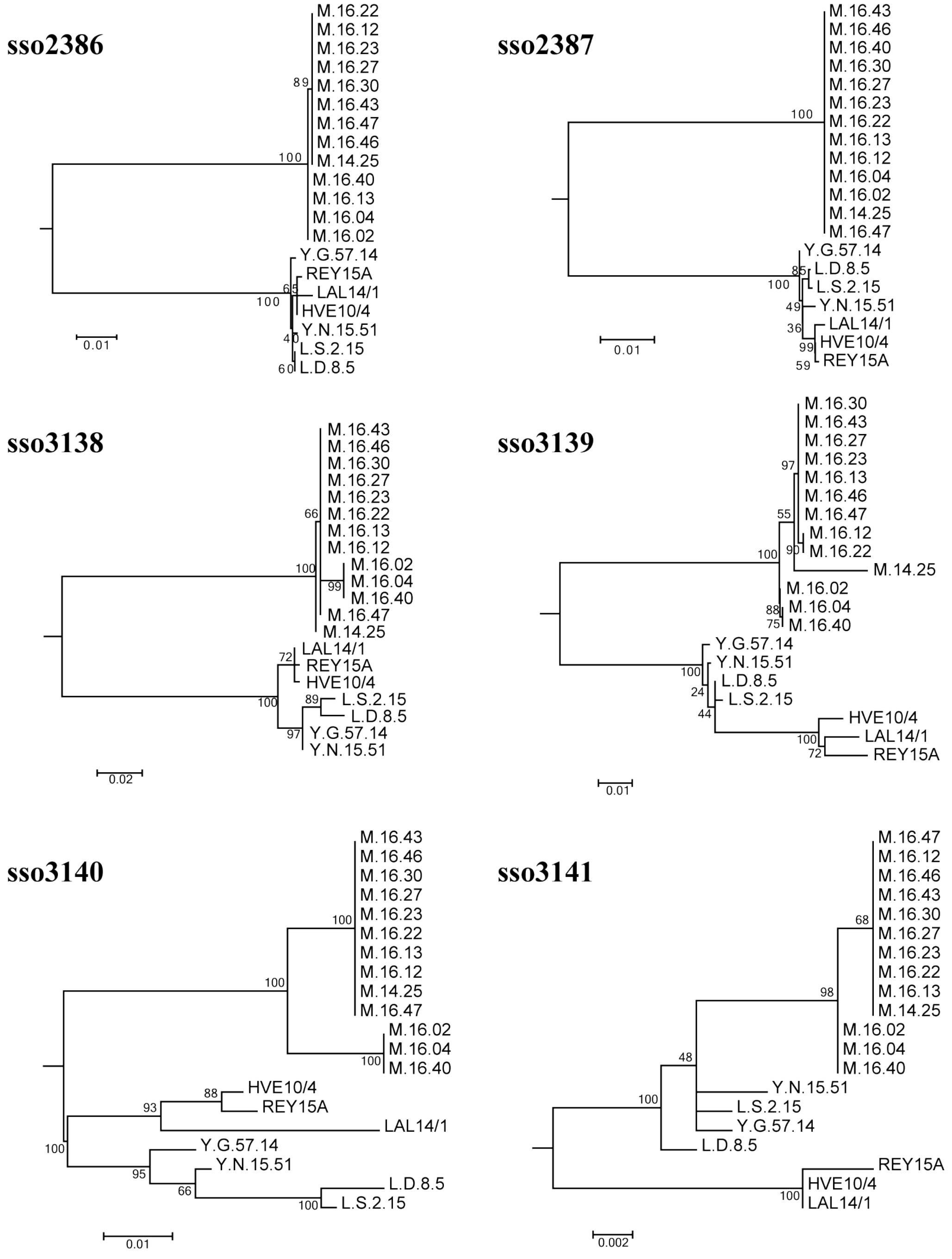

Figure S6. Maximum Likelihood phylogenies of *Sulfolobus islandicus* gene homologs of genes implicated in SIRV resistance in *Sulfolobus solfataricus*. Numbers at branches indicate bootstrap support values from Maximum Likelihood as a percent of 100 replicates. Scale bar represents substitutions per site.
